# High Resolution Population Distribution Maps for Southeast Asia in 2010 and 2015

**DOI:** 10.1371/journal.pone.0055882

**Published:** 2013-02-13

**Authors:** Andrea E. Gaughan, Forrest R. Stevens, Catherine Linard, Peng Jia, Andrew J. Tatem

**Affiliations:** 1 Department of Geography, University of Florida, Gainesville, Florida, United States of America; 2 Emerging Pathogens Institute, University of Florida, Gainesville, Florida, United States of America; 3 Land-use Environmental Change Institute, University of Florida, Gainesville, Florida, United States of America; 4 Fogarty International Center, National Institutes of Health, Bethesda, Maryland, United States of America; 5 Fonds National de la Recherche Scientifique, Brussels, Belgium; 6 Biological Control and Spatial Ecology, Université Libre de Bruxelles, Brussels, Belgium; University of Catania, Italy

## Abstract

Spatially accurate, contemporary data on human population distributions are vitally important to many applied and theoretical researchers. The Southeast Asia region has undergone rapid urbanization and population growth over the past decade, yet existing spatial population distribution datasets covering the region are based principally on population count data from censuses circa 2000, with often insufficient spatial resolution or input data to map settlements precisely. Here we outline approaches to construct a database of GIS-linked circa 2010 census data and methods used to construct fine-scale (∼100 meters spatial resolution) population distribution datasets for each country in the Southeast Asia region. Landsat-derived settlement maps and land cover information were combined with ancillary datasets on infrastructure to model population distributions for 2010 and 2015. These products were compared with those from two other methods used to construct commonly used global population datasets. Results indicate mapping accuracies are consistently higher when incorporating land cover and settlement information into the AsiaPop modelling process. Using existing data, it is possible to produce detailed, contemporary and easily updatable population distribution datasets for Southeast Asia. The 2010 and 2015 datasets produced are freely available as a product of the AsiaPop Project and can be downloaded from: www.asiapop.org.

## Introduction

The global human population is projected to increase from 7 billion to over 9 billion between 2011 and 2050, with much of this growth concentrated in low income countries [1]. The greatest concentration in growth is set to occur in urban areas, disproportionately impacting Asia where half of the population is expected to be living in urban areas by 2020 [1]. The effects of such rapid demographic growth are well documented, influencing the economies, environment and health of nations [2]. To measure the impact of this population growth there is a need for accurate, spatially-explicit, high resolution maps that correctly identify population distributions through time.

While high-income countries often have extensive mapping resources and expertise at their disposal to create accurate and regularly-updated spatial population databases, across the lower income regions of the world relevant data are often either lacking or are of poor quality [3]. Since the 1990s there has been increasing interest in creating spatially-explicit, large-area gridded population distribution datasets [4], [5], [6] to support applications such as disease burden estimation, epidemiological modelling, climate change and human health adaptive strategies, disaster management, accessibility modelling, transport and city planning, poverty mapping and environmental impact assessment [5], [6], [7], [8], [9], [10]. Current global gridded population datasets that are freely available include the Gridded Population of the World (GPW) database, versions 2 and 3 [11], [12] and the Global Rural Urban Mapping Project (GRUMP) [13]. In addition, the LandScan Global Population database is updated annually, but has some access restrictions [14], [15], and the United Nation Environment Programme (UNEP) has compiled gridded datasets for Latin America, Africa, and Asia [16], [17], [18], while the AfriPop project provides freely-available gridded population data for Africa [6], [10], [19].

These datasets vary in their modelling techniques and the types of input data used for their construction [20]. Briefly, GPW employs an areal weighting technique that assumes uniformity in population distribution within each administrative unit [5]. The GRUMP dataset builds on the GPW approach, but incorporates satellite night-light derived urban-rural designations in the spatial reallocation of population for each census block [5]. LandScan, UNEP and AfriPop all use dasymetric modelling approaches, utilizing ancillary data, such as land cover, to refine and weight population densities. The LandScan method uses coefficient weights derived from a combination of land cover, transport network and topographic data to re-distribute census data in a gridded format [14], while the AfriPop Project [21] relies principally on land cover, climate zone and detailed settlement information for deriving census data redistribution weights within administrative units [10], [20].

Each dataset suffers from limitations for the Southeast Asia region, however, stemming from the input data, mapping process or data availability. While efforts have been focussed in the past on obtaining the most detailed and recent input census data for GPW and GRUMP construction, each remains based upon the circa 2000 round of censuses [5], [13], and are thus increasingly outdated. Similarly, the UNEP datasets are based on even older and less detailed input census data. Moreover, the mapping approaches used for the GPW and UNEP datasets have been shown to be generally less precise than that undertaken for GRUMP, LandScan and AfriPop [6], [10], [22]. While an improvement, GRUMP datasets utilise satellite night time light-derived urban extents that have been shown to overestimate actual urban extents for large cities, while missing smaller settlements [23], [24], [25]. Finally, LandScan does not release information on the input demographic and ancillary spatial datasets, nor does it provide details on modelling methods, making assessments of its accuracy, reproducibility and judgements on its suitability difficult or impossible.

In this study we follow a similar approach utilised by the AfriPop Project [26]. We apply a model based on measured relationships between land cover and population density [20] to redistribute administrative unit populations to grid cells in Southeast Asia. We define the region using the official designation of the Association of Southeast Asian Nations (ASEAN) and include Timór-Leste for spatial contiguity (Table 1).The approach includes separation of urban and rural settlement extents and the integration of remotely sensed land cover data [10], [27]. It is important to identify urban from rural areas as the difference in population densities necessitates different land cover weights for distributing population across the landscape. In addition, demographic characteristics and urban versus rural growth rates make it important to treat urban areas different from settlement extents in the redistribution of population. Final products are compared to derived datasets of other global population datasets to assess the accuracy of the different mapping techniques.

**Table 1:** Summary information on input population count data used in the construction of the AsiaPop datasets.

Country	Admin level used	Admin level name	No. admin units	Ave. spatial resolution (km)	Ave. no. people per admin unit (thousands)	Year of census/official estimate (E) data used	Population data source
Brunei Darussalam	2	Mukim	32	13	12	2011	Department of Statistics, Dept. of Economic Planning and Development, Brunei
Cambodia	3	Village	1621	11	8	2008	National Institute of Statistics, Cambodia
East Timor	1	District	13	34	82	2010	Direccao Nacional de Estatistica, Timore-Leste
Indonesia	4	Village	440	66	540	2010	Biro Pusar Statistik, Indonesia
Lao People’s Democratic Republic	1	Province	18	113	343	2009 (E)	Lao Department of Statistics, Laos
Malaysia	2	Administrative district	160	46	172	2010	Department of Statistics, Malaysia
Myanmar	2	District	314	46	162	2002 (E)	Department of Population, Myanmar/Digital Agricultural Atlas, FAO
Philippines	3	Municipality	1,647	13	54	2007	National Statistics Office, Philippines
Singapore	2	Planning area	5	11	753	2010	Statistics Singapore
Thailand	1	Changwat	78	81	805	2006	National Statistical Office Thailand
Vietnam	2/4	Commune/Tinh	63/10613	73/6	1363/7	1999/2009	General Statistics Office, Vietnam

The average spatial resolution is a country’s administrative unit “cell size,” calculated as the square root of the mean administrative unit area (adapted from Balk et al., 2004 [11]) while people per administrative unit was calculated by taking the total population and dividing it by the total number of administrative units for each country.

## Materials and Methods

### Population Count Data

Population count data were obtained for each country listed in Table 1, principally derived from national population and housing censuses, matched to GIS administrative boundaries for the latest round of censuses, and at as fine an administrative unit level as publicly available. Where the census data are over a decade old, official population estimates were used. Table 1 details the features of the demographic data used.

### Land Cover Data

Fine-scale, satellite imagery-derived land cover datasets were used to reallocate contemporary census-based spatial population count data. Land cover classes were based principally on the MDA GeoCover Land Cover Thematic Mapper (TM) database, a product that provides a consistent global mapping of 13 land cover classes derived from circa 2005, 30 meter spatial resolution Landsat TM spectral reflectance data [28]. The GeoCover imagery classes were reformatted to be consistent with the GlobCover designations used for AfriPop [20], reclassifying and resampling the data to 8.33×10^−4^ degrees spatial resolution (approximately 100 meters at the equator). For areas that were classified as cloud, shadow, or “No Data” we filled the data using the nearest neighbour algorithm to create a complete, void-filled land cover dataset for each country.

Additional country-specific datasets were used where available to refine the mapping of settlements and land cover. For Cambodia, land cover was refined using detailed water bodies and built area extent datasets from the Ministry of Land Management, Urban Planning, and Construction. For the Philippines and Myanmar, land cover was refined using detailed built area datasets from the Pacific Disaster Center, Global Hazards Information Network [29]. Building and residential data classes from OpenStreetMap (OSM) (http://download-int.geofabrik.de/osm/asia/) [30], [31], an open source product that provides free world-wide geographic datasets, were used to refine urban and rural settlement extents for all countries where it was available. Lastly, the GeoCover data does not differentiate large high density urban areas from smaller rural settlements so, for all countries, we applied a conditional statement that used the urban designations set by the GRUMP urban extents dataset to identify which built areas were ‘urban’ while all other built areas were classified as rural [13]. The inclusion of these additional steps to refine the original GeoCover land cover dataset provide a final product with the most updated ancillary information available on settlement and built landscape features included in the land cover input layer for modelling human population distribution at regional to continental scales. The final land cover datasets were comprised of nine land cover types. Analyses were conducted principally in ArcGIS 10.0 [32] and ERDAS Imagine 2011 [33].

### Population Distribution Modelling

To model population distributions for the Southeast Asia region, we adopted the methodology used in the construction of the AfriPop datasets [6], [7], [10], [20] and is detailed in Linard, et al. [6] (Text S1). Modifications to the process for Southeast Asia mapping included a change in the input land cover data by using the GeoCover dataset. We also adjusted land cover specific weightings to re-allocate population densities based on Asian climates and countries. An updated Köppen-Geiger classification was used, broken down into seven main climate zones [34]. Equatorial (Zone A) and Arid (Zone B) climates were separated into sub-zones based on precipitation, creating two categories for each zone (Table S1 in Text_S1).

**Figure 1:**
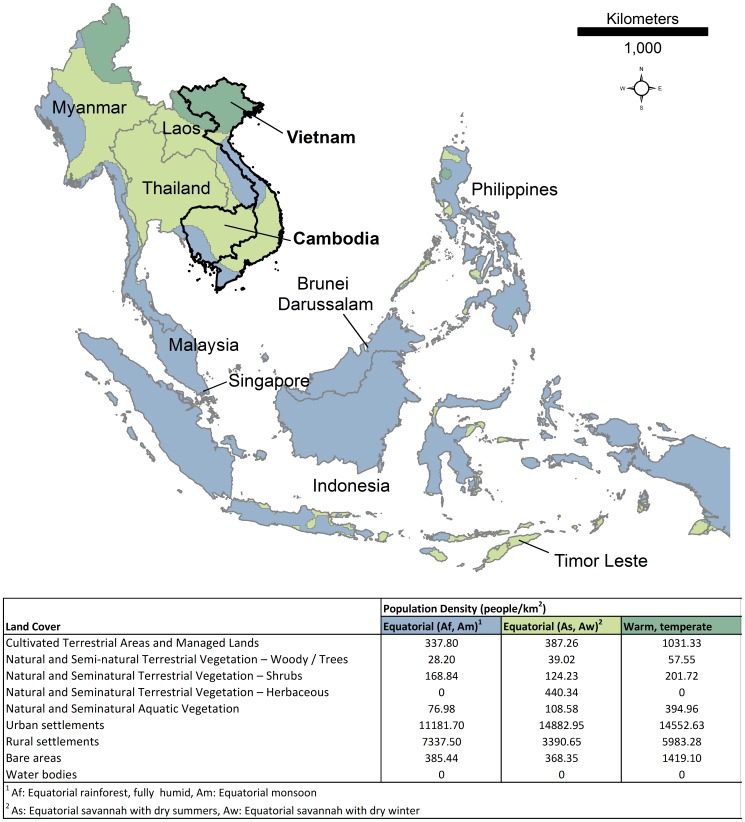
Köppen-Geiger climate classification [**34**] and associated land cover population density weightings for the Southeast Asia region.

As outlined in Linard, et al. [6], different sets of population densities were calculated on a pixel-by-pixel basis within each administrative unit based on the association between land cover and different climate zones. For Southeast Asia, the more detailed census data for Cambodia and Vietnam provided the input for generating per-land cover class population densities (Figure 1). Data from both countries were used to create per-climate zone average land cover-specific population density weightings which were then applied to redistribute rural populations within administrative units for all countries in Southeast Asia. The total population size at the national level was projected to 2010 and 2015 based on rural and urban growth rates estimated by the UN [1] using the following equation:
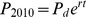
where *P_2010_* (*P_2015_*) is the required 2010 (2015) population, *P_d_* is the population at the year of the input population data, *t* is the number of years between the input data and 2010 (2015), and *r* is the urban or rural average growth rate taken from the UN World Urbanization Prospects Database, 2011 version (UNPD) [1]. We chose to use the more commonly used and publically available estimate values from the UNPD over alternative options [35] for consistency and standardization purposes to previous mapping efforts in Africa [6], [10], [19]. To assign respective growth rates, urban and rural areas were separated using the GRUMP urban extent dataset [5] by recoding any units to “rural units” if they did not spatially coincide with the GRUMP urban extent. Two versions of the datasets were produced, one with the total population adjusted to match UN national estimates [1], and the other left unadjusted.

### Accuracy Assessment

Since spatially detailed census data for Cambodia and Vietnam were available to facilitate modelling, these countries were also used in assessing the accuracy of the model. We aggregated the small administrative units to a coarser administrative unit level by summing the smaller units (Text S1, Figure S1). We then used these coarser units and population sums to generate gridded population maps and then compared sums of those gridded estimates with numbers from the original, fine-scale administrative unit populations. We compared the modelling method described above (referred to here as AsiaPop) to the methods used by two widely-used global population datasets, the Gridded Population of the World (GPW version 3) and the Global Rural Urban Mapping Project (GRUMP version 1). The original datasets are both available from the Center for International Earth Science Information Network (CIESIN) at Columbia University. Since we were interested in comparing the accuracies of the modelling processes, not that of the final derived products, we replicated the methodologies used in constructing GPW and GRUMP to ensure that identical input data were used for a fair comparison. GPW and GRUMP comparisons were chosen because these datasets have transparent, easily reproducible methods that are well documented [5], [11], [13], [36]. We did not include the UNEP methods and datasets in the comparison due to the relatively old age of the data, nor did we include LandScan due to a lack of published information on data sources and the modelling approach used [19]. Comparisons were conducted by aggregating the finest available census population counts (Admin Level 3) to the next level coarser (Admin Level 2). We then used those counts to produce gridded population distributions at 8.33×10^−4^ degrees spatial resolution using each of the three methods and compared the observed population totals at the finer administrative level with the summed estimates from the output gridded datasets at the coarser level.

Statistical analyses for observed and estimated population counts included measures of squared error and the Kruskal-Wallis test. The Kruskal-Wallis test is a non-parametric alternative to a one-way ANOVA test [37] and was necessary due to the non-normal distribution of counts. Post-hoc results for the Kruskal-Wallis test were employed in pairwise comparisons [38] and were done using the *pgirmess* [39] package in R 2.15.1 [40].

## Results

**Figure 2.The:**
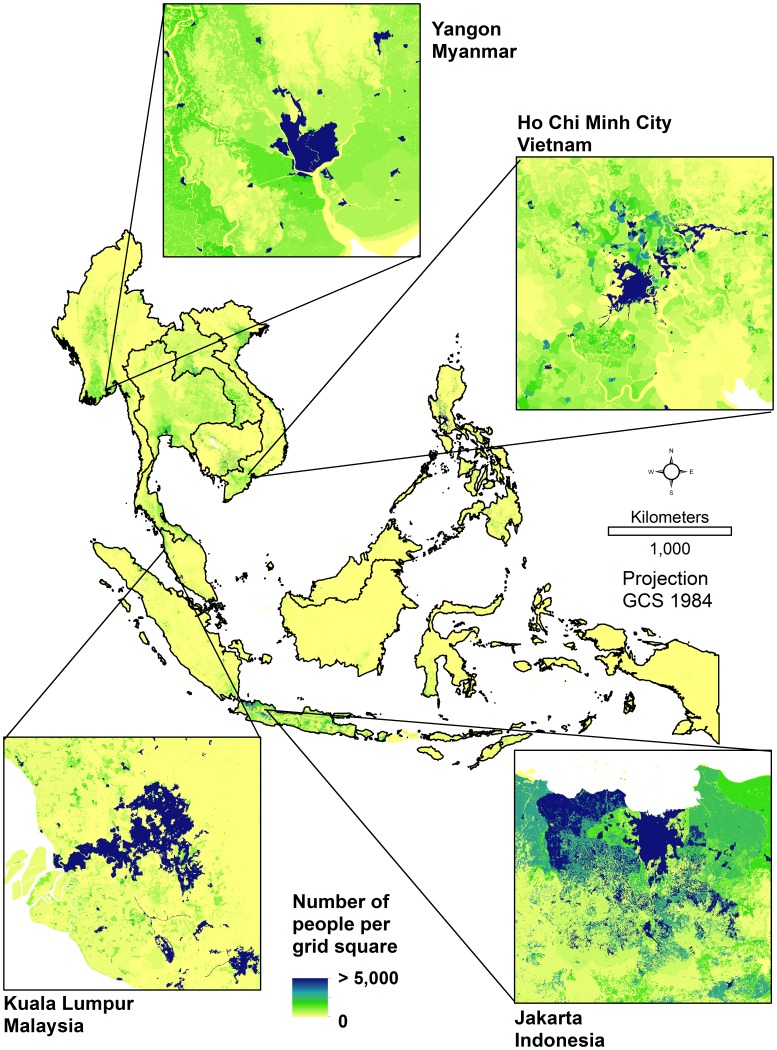
modelled spatial distribution of population in Southeast Asia, 2015. Output datasets are produced in geographic projection with square grid squares that are 8.33×10^−4^ degrees on a side (∼100 meters at the equator).

Population datasets for 2010 and 2015, non-adjusted and adjusted to UN national total estimates [1], were generated at 8.33×10^−4^ degrees spatial resolution and projected to a geographic coordinate system and WGS 84 datum for the ten Southeast Asian nations. Figure 2 shows the projected 2015 population distribution for Southeast Asia, displaying number of people per grid cell (8.33×10^−4^ degrees). Areas highlighted are some of the largest cities in the region and their surroundings.

### Accuracy Assessments

**Figure 3:**
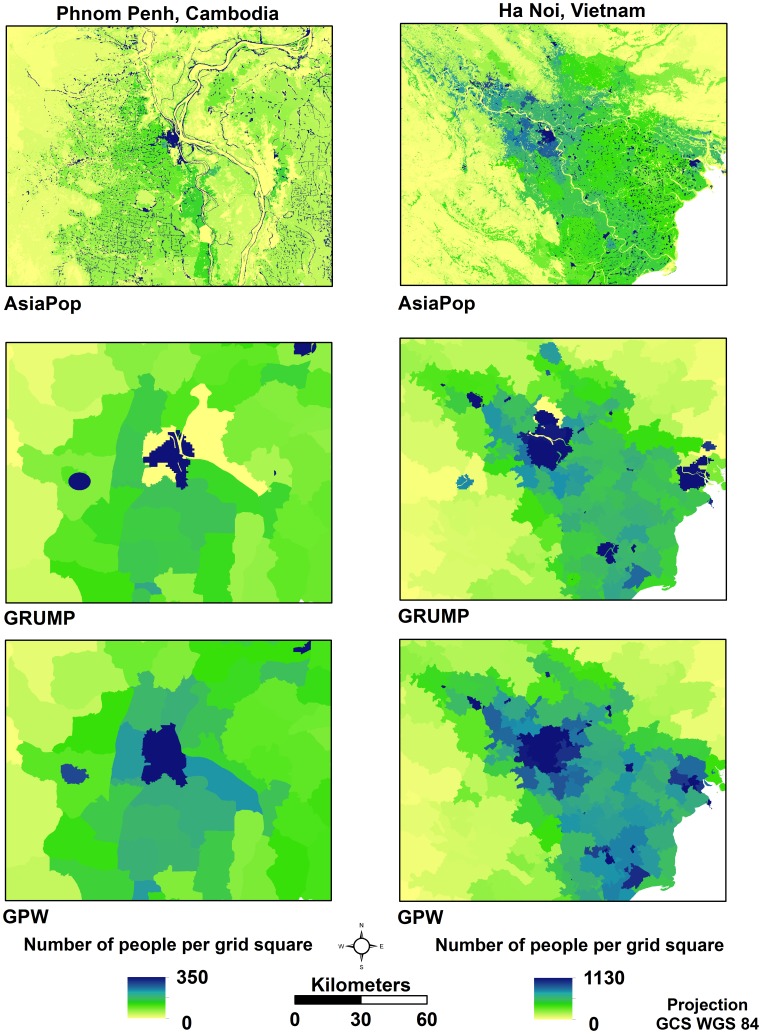
Visual comparisons are made of the AsiaPop, GRUMP v1, and GPW v3 gridded population datasets for Phnom Penh, Cambodia and Hanoi, Vietnam. These datasets were generated using census data one level coarser than the finest detail available.

Population distribution datasets were constructed using AsiaPop, GRUMP v1, and GPW v3 methodologies using Level 2 census data for Cambodia (census year: 2008) and Vietnam (census year: 1999). Figure 3 shows the modelled outputs, focused on two major urban centres, Phnom Penh, Cambodia and Ha Noi, Vietnam. Visual comparison of the three datasets highlights the underlying approach used for each one. GPW v3 evenly distributes the population across each individual administrative unit while GRUMP concentrates the population into a few major urban areas and then uses areal weighting to redistribute the remainder of the population [13]. AsiaPop also concentrates the population in settlements (defined using Landsat-derived land cover) but, in addition, weights population outside settlements based on different land cover types.

**Figure 4:**
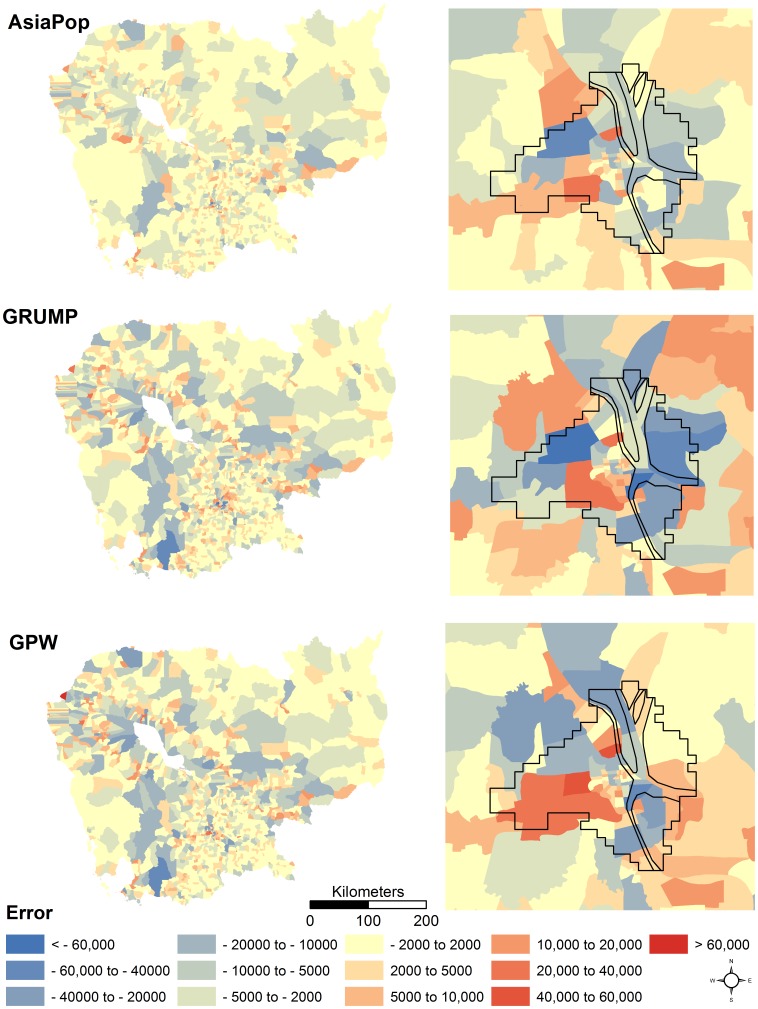
Errors mapped for population distribution datasets produced using the AsiaPop, GRUMP v1, and GPW v3 methodologies for a) Cambodia and b) Phnom Penh, Cambodia. Populations underestimated are highlight in blue and overestimated values are shown in red. The white outline highlights the boundary of Phnom Penh (source: GRUMP urban extent dataset).

The absolute error of the different population maps for Cambodia is shown in Figure 4. The AsiaPop method, in general, produces more accurate results, with many more administrative units showing low error values compared to the GRUMP and GPW methodologies.

**Figure 5:**
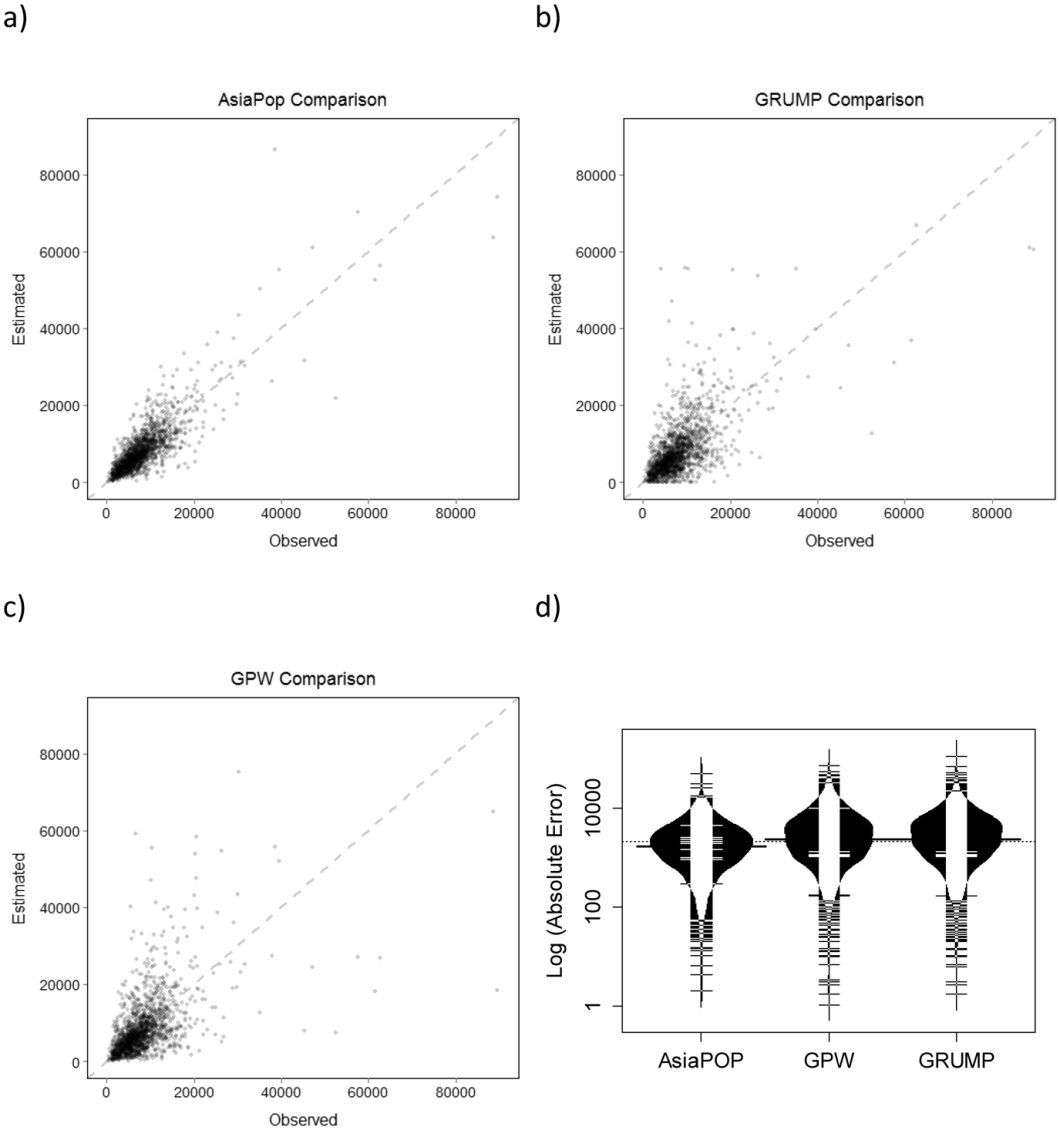
Scatter plots of observed, level 3 census data plotted against estimated modelled data from 2008. Data for Cambodia are shown for the a) AsiaPop, b) GRUMP, and c) GPW methods. In the d) bean plot, the distribution of mean absolute error for Cambodia population estimates is plotted for all three model types. The median is indicated by the dark black line and the y-axis is log transformed. Each horizontal line represents an individual observation and the underlying black histogram indicates the spread of observations for each model type.

To compare the different population distribution modelling approaches we calculated root mean square error (RMSE), the percentage of RMSE, and the mean absolute error (MAE) (Table 2). For both Cambodia and Vietnam, the AsiaPop RMSE and MAE measures were lower than those for GRUMP v1 or GPW v3 datasets. The AsiaPop approach also produced the lowest difference between the RMSE and MAE values, suggesting that the variance in individual errors was less for this method than either the GRUMP v1 or GPW v3 methods. Figure 5 shows the relationship between estimated and observed population counts for Cambodia. Each point represents an estimated and actual population count for a level 3 administrative unit. The relationship between the predicted gridded estimates and the observed population totals is substantially more linear for the AsiaPop method than either the GRUMP v1 or GPW v3 method. The AsiaPop model also shows the highest correlation between estimated and observed values at 0.83 compared to the GRUMP v1 (0.62) and GPW v3 (0.53) methods. The fourth plot is a “bean” plot [41], which shows the variation of mean absolute error for each population model. Each independent sample (an administrative unit), is represented by the spread of the short, horizontal bars above and below the median (dark horizontal line) and the distribution for each model is shown with vertical histograms. Results for Vietnam do not show as strong a relationship (see Text S1, Figure S2) between estimated and observed population counts, although AsiaPop still shows the highest correlation at 62.2% versus 43.4% and 44.7% for GRUMP v1 and GPW v3 respectively. Results from the Kruskal-Wallis test indicate significant differences between population estimates (p-values <0.0001) for comparisons of the AsiaPop dataset to the datasets produced using the GRUMP v1 and GPW v3 methods for both countries. There was no statistically significant difference between mean ranks of GRUMP v1 and GPW v3 population estimates for either the Cambodia or Vietnam datasets.

**Table 2:** Accuracy assessment results for the AsiaPop, GRUMP and GPW modelling methods for a) Cambodia and b) Vietnam.

a.	Cambodia	RMSE	% RMSE	MAE
	**AsiaPop**	3834.51	46.40	2494.32
	**GRUMP**	6767.39	81.89	3889.39
	**GPW**	6794.88	82.22	4021.41
**b.**	**Vietnam**	**RMSE**	**% RMSE**	**MAE**
	**AsiaPop**	4943.31	70.13	3007.04
	**GRUMP**	6523.77	92.56	3771.64
	**GPW**	7081.76	100.47	3844.47

Two different error assessment methods are presented: root mean square error (RMSE), also expressed as a percentage of the mean population size of the administrative level (% RMSE); and the mean absolute error (MAE).

## Discussion

The need for spatially-explicit, large-scale mapping of human population distribution continues to grow, especially given the increasing demand and use of digital, open-access, global-scale datasets [19]. In this study, we present the approaches used to construct a more accurate and detailed population distribution dataset for Southeast Asia, a region that has seen a population increase of greater than 30% over the past 20 years [1], [2] influencing economic well-being, environment and health issues, and land use transformations [2], [42]. Comparison with alternative population distribution modelling approaches suggests that the AsiaPop dataset more accurately characterizes population distribution in the region than other existing global datasets (Figures 5 and Text S1, Figure S2).

The Southeast Asia gridded population dataset presented here takes advantage of the growing collection of open source spatial data of relevance to population distributions (e.g. OpenStreetMap [31]), combining them with remotely-sensed settlement and land cover data to more accurately map human population distributions at a finer spatial scale than ever before [10], [13], . As shown in previous studies, the use of fine-scale spatial units for census input data can reduce the level of error in the modelling process [20], [22], [43]. Additionally, weighting the distribution of a population by different land cover types, especially through incorporating detailed datasets on settlement and built areas, provides a more accurate representation of patterns of population density [7], [10].

While it remains difficult to validate large-scale population distribution datasets, given that no independent sources exist at a global scale [19], the accuracy of population maps can be assessed if there exists a reference dataset at a finer spatial resolution than maps generated [10], [15], [44], [45]. Using different administrative levels, we have shown here that the AsiaPop method was the most accurate modelling method for the redistribution of population counts compared to existing replicable approaches. The lower RMSE (Table 2) of the AsiaPop method indicates a better overall fit of the model. The smaller difference between RMSE and MAE values for the AsiaPop method suggests this approach also has less variability in errors.

The improvements in accuracies over existing mapping methodologies shown here are promising, but sources of error and uncertainty remain in the outputs that should be acknowledged in data usage. Input data error is an important source of potential uncertainty and differs across census datasets, especially in Southeast Asia where it is difficult to provide firm estimates on the number of people who practice swidden cultivation [46]. Further, the variety in ages and administrative unit levels of the input data used across the region (Table 1) means that mapping accuracies are likely different from country to country, where, for example, using Admin level 2 estimates from 2002 likely produces a substantially more uncertain output 2010 population distribution dataset than for Indonesia, where admin level 4 census data from 2010 is used. Moreover, grouping population numbers into different sized administrative units contributes to the modifiable areal unit problem, a source of error prevalent in analyses that use population parameters [47]. Datasets that vary in their spatial resolution can influence the reliability of statistical population estimates, with smaller units generating less reliable estimates but larger spatial units masking relevant geographic variation [48]. Another source of uncertainty stems from the fact that the urban and rural growth rates used here for temporal population projection are only national-scale and thus mask any sub-national variations occurring. Lastly, the use of broad climate zone categories to calculate land cover weightings, as was undertaken here, may not be as accurate a methodology as using multiple fine-scale spatial datasets on demographic, land use, topographic and infrastructure variables known to correlate with population distributions [6].

Future work will aim to exploit the wealth of spatial datasets on infrastructure, settlement locations, internally displaced populations and land use that are becoming increasingly available, especially for resource poor countries. We will employ a more sophisticated, regression tree-based approach to further improve the accuracy of output population distribution datasets and enable rapid updates. Moreover, the lack of large-area sub-national datasets on age and sex structures of populations is proving detrimental to many areas of research [49], and the derivation of specific age and sex group large area population distribution datasets built from census and household survey data will be a priority. The mapping here of settlements at a single point in time that are then used as inputs to 2010 and 2015 datasets likely does not produce the realistic patterns of change that occur through urban growth, thus, novel model-based approaches to simulating growth in urban extents are being developed to provide more realistic inputs to projected population mapping. Finally, geostatistical interpolation approaches developed elsewhere [50] are being adapted to exploit the increasing availability of geo-located household survey data [49] to produce statistically robust gridded datasets representing a range of demographic and health metrics.

Given the speed with which population growth and urbanisation are occurring across much of Southeast Asia, and the impacts these are having on the economies, environments and the health of nations, this study outlines a timely and relevant approach for providing national level population distribution data. Additionally, the Southeast Asian region has a range in spatially-detailed census aggregations providing a good basis for further testing and validation of the dasymetric modelling approach that relies on relationships between land cover and population density to redistribute population distribution in a spatially-explicit manner. The approach was designed with an operational application in mind, using simple and semi-automated methods to produce easily updatable maps as new censuses and ancillary datasets become available. Population datasets for 2010 and 2015 are freely available as a product of the AsiaPop Project and can be downloaded from the project website: www.asiapop.org.

## Supporting Information

Figure S1 **Different levels of administrative units, with a) finer census data accessed for Cambodia (level 3) and b) coarser (level 2) administrative units in which population totals were summed to for use in accuracy assessments.** The different coloured polygons in *b* simply identify boundaries of the finer scale census blocks defined by level 3 data while the darker lines outline the coarser, level 2 boundaries.
(TIF)

Figure S2 **Scatter plots of observed, level 3 census data plotted against estimated modelled data from 1999 for Vietnam for a) AsiaPop, b) GRUMP, and c) GPW.** In the d) bean plot, the distribution of mean absolute error for VNM population estimates is plotted for all three model types. The median is indicated by the dark black line and the y-axis is log transformed. Each horizontal line represents an individual observation and the underlying black histogram indicates the spread of observations for each model type.
(TIF)

Table S1 **The Köppen-Geiger climate classification (modified from Kottek et al., 2006), with sub-regions broken down for Zones A and B.** The criteria for sub-zones are based on precipitation and temperature minimums, annual totals and thresholds aggregated into a gridded dataset [1].
(DOCX)

Text S1 **High resolution population distribution maps for Southeast Asia in 2010 and 2015.**
(DOCX)
