# Supplementary figures and images for: High Resolution Population Distribution Maps for Southeast Asia in 2010 and 2015
Source: PLoS One. 2013 Feb 13;8(2):e55882. doi: 10.1371/journal.pone.0055882 (PMC3572178; doi:10.1371/journal.pone.0055882)

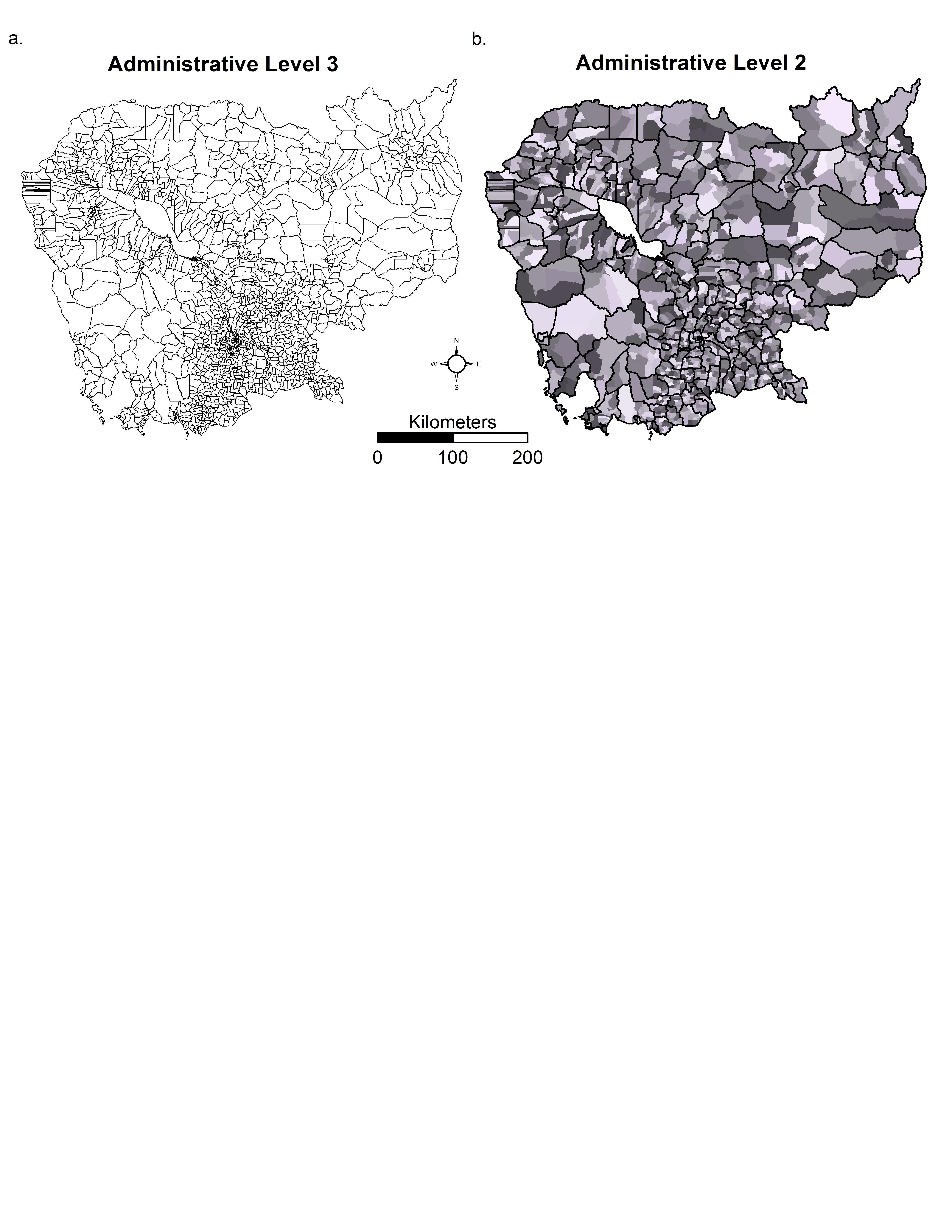

Supplement: Figure S2 — Scatter plots of observed, level 3 census data plotted against estimated modelled data from 1999 for Vietnam for a) AsiaPop, b) GRUMP, and c) GPW. In the d) bean plot, the distribution of mean absolute error for VNM population estimates is plotted for all three model types. The median is indicated by the dark black line and the y-axis is log transformed. Each horizontal line represents an individual observation and the underlying black histogram indicates the spread of observations for each model type. (TIF) [file pone.0055882.s002.tif]
