# Supplementary material for: High Resolution Population Distribution Maps for Southeast Asia in 2010 and 2015
Source: PLoS One. 2013 Feb 13;8(2):e55882. doi: 10.1371/journal.pone.0055882 (PMC3572178; doi:10.1371/journal.pone.0055882)
Supplement: Table S1 — The Köppen-Geiger climate classification (modified from Kottek et al., 2006), with sub-regions broken down for Zones A and B. The criteria for sub-zones are based on precipitation and temperature minimums, annual totals and thresholds aggregated into a gridded dataset [1]. (DOCX) [file pone.0055882.s003.docx]

| **Zone Name** | **Köppen-Geiger Types** | **Description** |
| --- | --- | --- |
| A1 | Af, Am | Equatorial rainforest, fully humid, Equatorial monsoon |
| A2 | As, Aw | Equatorial savannah with dry summer, Equatorial savannah with dry winter |
| B1 | BS | Arid, steppe climate |
| B2 | BW | Arid, desert climate |
| C | Cs, Cw, Cf | Warm temperate climates |
| D | Ds, Dw, Df | Snow climates |
| E | ET, EF | Polar climates |
