# Supplementary material for: High Resolution Population Distribution Maps for Southeast Asia in 2010 and 2015
Source: PLoS One. 2013 Feb 13;8(2):e55882. doi: 10.1371/journal.pone.0055882 (PMC3572178; doi:10.1371/journal.pone.0055882)
Supplement: Text S1 — High resolution population distribution maps for Southeast Asia in 2010 and 2015. (DOCX) [file pone.0055882.s004.docx]

## Text S1: High resolution population distribution maps for Southeast Asia in 2010 and 2015

**1.1 Climate classifications for land cover weightings**

For calculating the land cover weightings used in the AsiaPop methodology, we used an updated Köppen-Geiger classification broken down into seven main climate zones [1]. Equatorial (Zone A) and Arid (Zone B) climates were separated into sub-zones creating two categories for each zone (Table S1). Zones are constructed using a combination of criteria that delineate biophysical conditions that are broadly comparable across large areas (Table S1) [1]. To create climate zone weightings for population redistribution a large number of administrative units within each zone is necessary. We chose to group the Köppen-Geiger classification types in a way that distinguishes varying biophysical conditions within Asian countries while still providing a large sample of administrative units within each zone.

**1.2 Data used in assessing the accuracy of population distribution models**

We compared the per-unit population counts between the fine resolution datasets (Level 3) and estimations summed from gridded outputs generated using the next, more aggregated level (Level 2) for AsiaPop, GPW v3, and GRUMP v1 methods. An example of the spatial extent of these two administrative levels is given in Figure S1. We then used the aggregated administrative units and their populations to produce gridded population distributions at 8.33 x 10^-4^ degrees spatial resolution using each of the three methods and compared the observed population totals at the finer administrative level with the summed estimates from the output gridded datasets. Census data across a range of administrative unit levels were collected for Cambodia (2008) and Vietnam (1999). Vietnam has more recent census data available (2009), for which AsiaPop product totals are scaled to match, but the available 2009 census data did not have as fine a resolution as the 1999 census data. Since we aggregate census data to a coarser resolution for accuracy assessment, we used the 1999 census data to preserve spatial accuracy for assessment purposes. For each level of aggregation, the final gridded product retains a spatial resolution of 8.33 x 10^-4^ degrees on a side (~100 meters at the equator).

**1.3 Accuracy assessment for Vietnam**

The relationship between observed and estimated modelled datasets for Vietnam has a weaker association than that shown in the main text for Cambodia (Figure S2). However, AsiaPop still has the highest correlation at 62.2% versus 43.4% and 44.7% for GRUMP and GPW respectively. The Vietnam observed census data (n = 10,613) includes very fine-scaled administrative units, many (n = 391) which have zero or close to zero population numbers. Administrative units having zero population and therefore zero error, were excluded when plotting the log absolute error for Vietnam using the "beanplot" distribution (Figure S1.3d).

**References**

1. Kottek M, Grieser J, Beck C, Rudolf B, Rubel F (2006) World map of the Koppen-Geiger climate classification updated. Meteorol Z 15: 259-263.
